# Role of microglia reactivity and sex on the cognitive decline linked to sporadic hypercholesterolemia

**DOI:** 10.1002/alz70855_105776

**Published:** 2025-12-23

**Authors:** Matheus Scarpatto Rodrigues, Natália Baltazar Nascimento, Gabriela Joras Baumart, Hemelin Resende Farias, Alessandra Gonçalves Machado, Lucas Santos, Tainá Schons, Alex Paulo Padilha, Mariana Viana Costa, Rachel KSS Bast, Daniel Pens Gelain, José Claudio Fonseca Moreira, David Engblom, Andreza Fabro de Bem, Jade de Oliveira

**Affiliations:** ^1^ Universidade Federal do Rio Grande do Sul, Porto Alegre, Brazil; ^2^ University of Pittsburgh, Pittsburgh, PA, USA; ^3^ Universidade Federal do Rio Grande do Sul (UFRGS), Porto Alegre, Brazil; ^4^ Departamento de Bioquimica, Universidade Federal do Rio Grande do Sul, UFRGS, Porto Alegre, RS, Brazil; ^5^ Institute of Biomedical Sciences, Universidade Federal do Rio de Janeiro (UFRJ), Rio de Janeiro, RJ, Brazil; ^6^ UFRGS, Porto Alegre, Brazil; ^7^ Universidade Federal do Rio Grande do Sul, Porto Alegre, Rio Grande do Sul, Brazil; ^8^ Linkoping University, Linkoping, Sweden; ^9^ Universidade de Brasília, Brasília, Brazil

## Abstract

**Background:**

High blood cholesterol levels are recognized as an important risk factor for dementia, including Alzheimer's disease (AD). Although the mechanisms linking high cholesterol to cognitive decline remain unclear, blood‐brain barrier dysfunction and neuroinflammation appear to connect cholesterol dishomeostasis to brain disorders. Previous studies have shown that elevated blood cholesterol levels can activate microglia, modulating its reactivity in the central nervous system. Therefore, this study aims to investigate the role of microglial reactivity in cognitive impairments associated with sporadic hypercholesterolemia.

**Method:**

To generate an animal model of sporadic hypercholesterolemia, male and female adult CF‐1 mice were fed a high‐cholesterol diet (2.5% of cholesterol and cholic acid 0.5%), whereas control mice were fed a normal diet for eight weeks. In the last four weeks, mice were concomitantly treated by oral gavage (once a day) with minocycline, a pharmacological modulator of microglia reactivity.

**Result:**

Mice fed a cholesterol‐rich diet for eight weeks showed a significant increase in total plasma cholesterol levels and impaired performance in hippocampal‐dependent memory tasks. Interestingly, minocycline treatment improved hypercholesterolemic mice performance in memory tasks. The memory deficits associated with sporadic hypercholesterolemia were accompanied by a decreased number of lectin‐positive cells and a reduced presence of microglia in the hippocampal perivascular area, a process that was not influenced by minocycline treatment or sex. Interestingly, minocycline treatment increased Claudin‐5 immunocontent in the prefrontal cortex and hippocampus of hypercholesterolemic mice. However, no significant changes in microglial density or morphology were observed in these brain regions, regardless of pharmacological intervention or sex.

**Conclusion:**

Taken together, our results demonstrate that sporadic hypercholesterolemia in mice was accompanied by deficits in hippocampal‐dependent memory tasks and a reduction in microglial presence in the perivascular area. Notably, minocycline treatment improved memory in hypercholesterolemic mice and increased Claudin‐5 immunocontent without altering microglial density or morphology or their presence in the perivascular area. No significant sex differences were observed, indicating that the effects of sporadic hypercholesterolemia in mice are consistent across sexes.

